# Enhancement of Power Conversion Efficiency of Non-Fullerene Organic Solar Cells Using Green Synthesized Au–Ag Nanoparticles

**DOI:** 10.3390/polym15061482

**Published:** 2023-03-16

**Authors:** Victor Okai, Habibat Faith Chahul, Rafi Shikler

**Affiliations:** 1 Department of Chemistry, Federal University of Agriculture, Makurdi P.M.B. 2373, Benue Sate, Nigeria; 2 School of Electrical and Computer Engineering, Ben Gurion University of the Negev, Beer-Sheva 8410501, Israel; 3 Ilse-Katz Nanocenter, Ben Gurion University of the Negev, Beer-Sheva 8410501, Israel

**Keywords:** organic solar cell, non-fullerene, green synthesis, onion-extract, Au–Ag, scattering

## Abstract

Organic-based photovoltaics are excellent candidates for renewable energy alternatives to fossil fuels due to their low weight, low manufacturing cost, and, in recent years, high efficiency, which is now above 18%. However, one cannot ignore the environmental price of the fabrication procedure due to the usage of toxic solvents and high-energy input equipment. In this work, we report on the enhancement of the power conversion efficiency non-fullerene organic solar cells by incorporating green synthesised Au–Ag nanoparticles, using onion bulb extract, into the hole transport layer poly (3,4-ethylene dioxythiophene)-poly (styrene sulfonate) (PEDOT: PSS) of Poly[4,8-bis(5-(2-ethylhexyl)thiophen-2-yl)benzo[1,2-b;4,5-b′]dithiophene-2,6-diyl-alt-(4-(2-ethylhexyl)-3 fluorothieno[3,4-b]thiophene-)-2-carboxylate-2-6-diyl)]: 3,9-bis(2-methylene-(3-(1,1-dicyanomethylene)-indanone))-5,5,11,11-tetrakis(4-hexylphenyl)-dithieno[2,3-d:2′,3′-d′]-s-indaceno[1,2-b:5,6-b′]dithiophene (PTB7-Th: ITIC) bulk-heterojunction organic solar cells. Red onion has been reported to contain quercetin, which serves as a capping agent that covers bare metal nanoparticles, thus reducing exciton quenching. We found that the optimized volume ratio of NPs to PEDOT: PSS is 0.06:1. At this ratio, a 24.7% enhancement in power conversion efficiency of the cell is observed, corresponding to a 9.11% power conversion efficiency (PCE). This enhancement is due to an increase in the generated photocurrent and a decrease in the serial resistance and recombination, as extracted from the fitting of the experimental data to a non-ideal single diode solar cell model. It is expected that the same procedure can be applied to other non-fullerene acceptor-based organic solar cells, leading to an even higher efficiency with minimal effect on the environment.

## 1. Introduction

High energy demand is one of the major issues of the unprecedented rate of population and economic growth [1]. Currently, over 80% of the world’s energy consumption is from fossil fuel. However, fossil fuel quantities are limited and cannot meet the world’s increasing energy demands [2]. Moreover, the environmental crisis generated using fossil fuel has attracted an increasing level of concern from the scientific community, engineering community, and government. Today, it is widely agreed that there is a need to generate energy from renewable and eco-friendly sources. The natural Earth’s source of energy is the sun, and the sun’s energy can be converted to electricity through the photovoltaic effect. Among various fields of photovoltaic technologies, the bulk-heterojunction organic solar cells (OSC) have been shown to be extremely promising candidates due to their low material cost, simple fabrication methods, and flexibility [3]. However, organic photovoltaic cells still have low power conversion efficiency (PCE) with respect to other competitive technologies. This is because the short exciton’s diffusion length, exciton quenching, and low carrier mobility of the active layer, resulting in low absorption efficiency [4]. Several efforts have been made to address this problem. The incorporation of metal nanoparticles in the solar cell’s architecture is one of the recent approaches. Table 1 summarizes some of the recent works in this area.

Gold and silver are well-known choices in the scattering and plasmonic field due to their resonance across the visible spectrum range and the developed free-electron densities [11,19,20]. The addition of Au, Ag, or both to organic solar cells has been carried out by several research groups. Alkhalayfeh et al. [7] incorporated Au–Ag NPs into a buffer layer (poly (3,4-ethylene dioxythiophene)): poly (styrene sulfonate) (PEDOT: PSS) and active layer (PTB7: PC71BM) to improve power conversion efficiency (PCE) from 2.50 to 4.24%. They reported that the coupled effects of nanoparticles positively influence the localized surface plasmon resonance (LSPR), therefore enhancing the absorption in the active layer [7]. Tran et al. embedded Au–Ag core-shell NPs in the PCPDTBT: PC60BM active layer, which increased the PCE from 0.0057 to 0.0396%. The PCE enhancement was attributed to the high scattering power of Ag NPs and the broad spectral response of Au NPs in the long-wavelength range [21]. Core-shell, metal nanostructures, bimetallic, and mixed metal nanoparticles have been incorporated in the buffer, active layer, or both to enhance absorption in a broad wavelength range [14,21]. Kacus et al. improved PCE from 2.11 to 2.55% by embedding Au NPs into the active layer; a 2.23% increase was reported using Ag NPs in the same active layer [10]. The authors suggested that PCE enhancement is because the Au NPs and Ag NPs are acting as light scattering centers in the active layer [10]. Rivera-Taco et al. enhanced the optical absorption by incorporating Ag NPs into the PEDOT: PSS. The study reported an increase in the short-circuit current density ($J_{\mathrm{SC}}$) and fill factor (FF) ($J_{\mathrm{SC}}$) to 15.5 $\mathrm{mA}/{\mathrm{cm}}^{2}$ and 0.49%, respectively, when compared to reference PEDOT: PSS with 15.4 $\mathrm{mA}/{\mathrm{cm}}^{2}$ and FF of 0.45% [8]. However, the limitation of incorporating NPs into the photoactive layer is that the NPs can become charge recombination centers that introduce exciton quenching and non-radiative energy interaction between the metal NPs and active layers, resulting in an overall reduction in efficiency [14,22]. All of these results were conducted with fullerene-based OSC. Peng et al. managed to enhance the efficiency of non-fullerene-based OSC using silver nanowires incorporated into the PEDOT: PSS [16]. These recent results are promising but an additional aspect that must be taken into account is the environmental cost of the synthesis route of these NPs. The need to employ environmentally benign procedures for the synthesis of metallic NPs has led researchers to the use of plants, algae, fungi, bacteria, and viruses for the production of low-cost, highly efficient, and non-toxic metallic NPs (see ref. [23] in the context of dye sensitized solar cells).

In this study, we demonstrate a green method of synthesizing metal nanoparticles using renewable and eco-friendly aqueous onion bulb extract instead of using toxic solvents and high-energy input equipment. Red onion has been reported to contain quercetin, which serves as a capping agent that covers bare metal nanoparticles [24,25], thus reducing exciton quenching. Moreover, proper volume ratios of metal nanoparticles are required to be incorporated in the buffer layer to ascertain an optimum measure in improving the PCE of the photovoltaic device. This study applies a method that uses the biosynthesize Au–Ag nanoparticles in the PEDOT: PSS to enhance light scattering effects in the photoactive layer. We study the effect an optimized device with Poly[4,8-bis(5-(2-ethylhexyl)thiophen-2-yl)benzo[1,2-b;4,5-b′]dithiophene-2,6-diyl-alt-(4-(2-ethylhexyl)-3 fluorothieno[3,4-b]thiophene-)-2-carboxylate-2-6-diyl)]: 3,9-bis(2-methylene-(3-(1,1-dicyanomethylene)-indanone))-5,5,11,11-tetrakis(4-hexylphenyl)-dithieno[2,3-d:2′,3′-d′]-s-indaceno[1,2-b:5,6-b′]dithiophene (PTB7-Th: ITIC) as the active layer, leading to a 24.7% power conversion efficiency (PCE) improvement.

## 2. Materials and Methods

### 2.1. Materials

Fresh onion bulb was bought from the urban market Beer sheva, Israel. Chloroauric acid (HAuCl_4_), silver nitrate(AgNO_3_), 3,9-bis(2-methylene-(3-(1,1-dicyanomethylene)-indanone))-5,5,11,11-tetrakis(4-hexylphenyl)-dithieno[2,3-d:2′,3′-d′]-s-indaceno[1,2-b:5,6-b′]dithiophene (ITIC), Poly[4,8-bis(5-(2-ethylhexyl)thiophen-2-yl)benzo[1,2-b;4,5-b′]dithiophene-2,6-diyl-alt-(4-(2-ethylhexyl)-3 fluorothieno[3,4-b]thiophene-)-2-carboxylate-2-6-diyl)] (PTB7-Th), Poly(9,9-bis(3′-(N,N-dimethyl)-N-ethylammoinium-propyl-2,7-fluorene)-alt-2,7-(9,9-dioctylfluorene))dibromide (PFN-Br), 1,8 Diiodooctane, and Poly(3,4-ethylenedioxythiophene)-poly(styrenesulfonate) (PEDOT: PSS 1.3 wt% dispersion in water) were purchased from Sigma-Aldrich, Israel. Chlorobenzene, acetone, isopropyl alcohol, ethanol, and deionized water were purchased from Bio-Lab ltd, Jerusalem, Israel, and Glass/ITO substrates with a sheet resistance of 20 Ω/sq from Xin Yan Technology Ltd., Hong Kong, China.

### 2.2. Preparation of Red Onion Extraction

A total of 25 g of fresh onion bulb was thoroughly washed in deionized water and finely crushed with a laboratory mortar. The crushed onion was mixed with deionized water (100 mL) in a 500 mL Erlenmeyer flask, then heated at 50 °C for 10 min. Finally, the broth was filtered with Whatman 201 filter papers (90 mm diameter), and the filtrate was kept in the refrigerator at 4 °C before its application in the synthesis of the nanoparticles [26].

### 2.3. Biosynthesis of Au–Ag Nanoparticles

For the synthesis of Ag–Au nanoparticles, 1 mM of 5 mL of HAuCl_4_ was added drop by drop to 90 mL of the extract and was stirred for 30 s. Then, a corresponding portion of the AgNO_3_ was added in the same manner and allowed to complete the reaction in the dark for 24 h with constant stirring.

The color of the solution changed from colorless to reddish-brown. The color change was a result of the bioreduction of Au^3+^-Ag^+^ metallic precursor to Au°-Ag° NPs. In addition to the change in color, UV–Vis analysis was carried out to confirm the formation of nanoparticles. This further proves that the method adopted in the synthesis is probably good for nanoalloy NPs synthesis. The approach was similar to the literature report but with some modifications [27].

#### Isolation and Purification of the Biosynthesized Au–Ag Nanoparticles

Centrifugation was used to isolate and purify the biosynthesized Au–Ag nanoparticles from the reaction mixture. The nanoparticle pellets were obtained from the reaction mixture by subjecting the mixture to centrifuge at 10,000 rpm for 20 min. For purification, the pellets were re-dispersed into the mixture of 70 mL of absolute ethanol and 30 mL of deionized water and centrifuged at the same rpm and time. This was carried out to remove unbounded organic molecules and reduce the insulating nature of the capping agent from the nanoparticle surface to be fit for its application in solar cells. Care must be taken to avoid the total removal of the capping agent, which may lead to aggregation of the nanoparticles. Subsequently, the nanoparticles were re-dispersed in deionized water and kept in the refrigerator at 4 °C before their use.

### 2.4. Organic Solar Cells Device Fabrication

PEDOT: PSS layers with different concentrations of biosynthesized Au–Ag NPs were prepared by adding 30 μL, 60 μL, and 90 μL of Au–Ag NPs colloidal solution into 1 mL of PEDOT: PSS (1.3 wt% dispersion in H_2_O). Before spin-coating on the glass-coated ITO substrates, the substrates were cleaned by sequential treatment with deionized water, acetone, and isopropanol in an ultrasonic bath for 15 min, then treated with oxygen plasma for 10 min, and the resulting solutions were ultra-sonicated for 35 min. A reference PEDOT: PSS and an ultra-sonicated mixture of Au–Ag NPs and PEDOT: PSS was spin-coated on top of the ITO layer at a speed of 2000 rpm for 60 s, followed by heating on a hot plate at 130 °C for 10 min to remove water (residual). The active layer’s solution was prepared by blending PTB7-Th: ITIC at 1:1.2 ratio in 970 μL of chlorobenzene and 30 μL of 1,8-diiodooctane with a total concentration of 25 mg/mL and stirring for 12 h in the glove box. The mixture was spin-coated on top of the reference PEDOT; PSS and PEDOT: PSS: Au–Ag NPs layer at the speed of 2500 rpm for 60 s. On top, 0.5 mg of PFN-Br in 1 mL of methanol at a speed of 2000 rpm for 60 s was spin-coated on the active layer film as an electron transport layer. At the rate of 0.5 Å/s, a 90 nm aluminum cathode thermally evaporated on top of the PFN-Br layer at a vacuum level of $2.8\;\times \;10^{-6}$ torr. To remove any residual water and organic polymer solvent, the organic solar cell was heated in the glove box at 130 °C for 10 min. The active area of the device was estimated to be 0.06 ${\mathrm{cm}}^{2}$.

### 2.5. Biosynthesize Au–Ag NPs and Organic Solar Cells Device Characterization

The biosynthesized Au–Ag nanoparticles (Au–Ag NPs) were characterized by transmission electron microscopy (TEM), X-ray diffraction (XRD), and UV–Vis spectroscopy. The biosynthesized nanoparticles’ structural and phase formation were characterized by X-ray diffraction (XRD) analysis. A UV–Vis spectrometer, JASCO (Model V-530, Mary’s Court Easton, MD, USA), with a wavelength range of 190–1100 nm, was used to measure the absorption of the colloidal nanoparticles in an optical quartz cuvette (10 mm length) and also for the measurement of the absorption of reference and ITO/PEDOT: PSS + Au–Ag/PTB7-Th: ITIC films at different concentrations. A transmission electron microscope (TEM), FEI Tecnai T12, with a high voltage range of 40–120 kV and high-resolution scanning electron microscope (HR-SEM) of FEI Quanta, FEG 200 make, was used to determine the shape, size, and morphology of the biosynthesized Au–Ag Nps. For the TEM, a well-dispersed aqueous nanoparticle was dropped on 300 carbon-coated meshes placed on a TEM grid. In SEM, PEDOT: PSS: Au–Ag NPs was coated on a substrate and placed on a SEM grid. At room temperature, the powder X-ray diffraction patterns of the biosynthesized Au–Ag NPs were recorded using a PANnalytical X’Pert PRO X-ray diffractometer with Cu Kα1 radiation (λ = 1.5405 Å) at a scanning rate of $2{\;\min}^{-1}$ and 2θ ranging from 20 to 80. For the device characterization, a solar simulator, Sciencetech SS150 (AAA type), was used, and the light intensity calibration was carried out at 100 $\mathrm{mW}/{\mathrm{cm}}^{2}$ using an Oriel reference cell. The Keithley 2636 source meter was used for the acquisition of J–V curves. Under the AM 1.5 spectrum provided by the solar simulator, the photovoltaic parameters—open circuit voltage ($V_{\mathrm{OC}}$), short circuit current density ($J_{\mathrm{SC}}$), fill factor (FF%), and power conversion efficiency (PCE%)—were determined from the J–V plots for the illuminated devices. Fitting the experimental data to the single diode model was conducted in MATLAB.

#### Parameters Characteristic of Solar Cells

Fill factor (FF), open-circuit voltage ($V_{\mathrm{OC}}$), power conversion efficiency (PCE), and short-circuit current density ($J_{\mathrm{SC}}$) are some of the parameters used to characterize solar cells. The PCE is defined by the ratio of the maximum power output to the input power:

(1) $$\mathrm{PCE}=\frac{J_{\mathrm{MPP}}{*V}_{\mathrm{MPP}}}{P_{\mathrm{in}}/\mathrm{area}}=\frac{J_{\mathrm{sc}}{*V}_{\mathrm{oc}}*\mathrm{FF}}{P_{\mathrm{in}}/\mathrm{area}}*100$$

where the FF is defined by the following relation [28]:

(2) $$\mathrm{FF}=\frac{J_{\mathrm{MPP}}{*V}_{\mathrm{MPP}}}{J_{\mathrm{sc}}{*V}_{\mathrm{oc}}}$$

## 3. Results and Discussion

### 3.1. Characterizations of Biosynthesized Gold-Silver Nanoparticles

**Figure 1:**
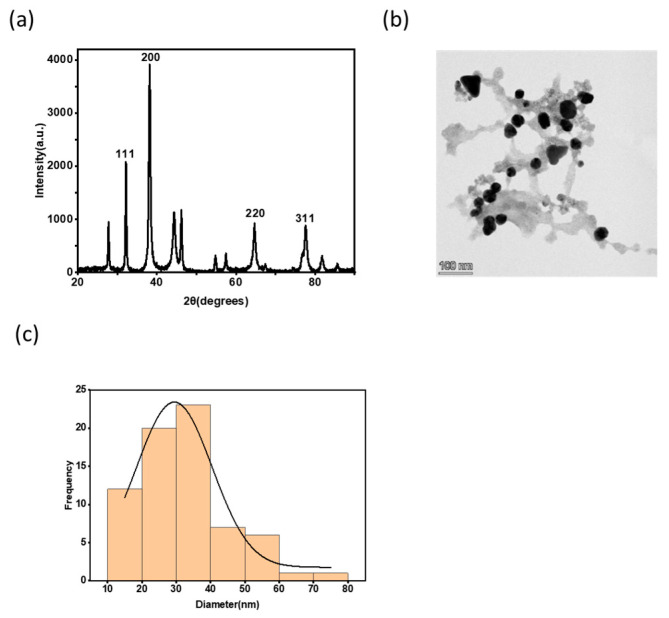
(**a**) XRD of the biosynthesized Au–Ag nanoparticles; (**b**) TEM image for the colloidal biosynthesized Au–Ag NPs solution; and (**c**) the Gaussian statistic size distribution of the nanoparticles (30 nm).

Figure 1a indicates the XRD pattern, and the four major characteristics of diffraction peaks for Au–Ag nanoparticles exhibited at 2θ angle of 32.2°, 38.2°, 64.7, and 77.6, respectively, were attributed to the lattice planes of (111), (200), (220), and (311), which confirm the crystalline nature and face-centered cubic (FCC) structure of Au–Ag NPs. Several reports [25], refs. [29,30,31] stated that red onions contain a flavonoid called quercetin which is responsible for the bio-reduction and the capping of the nanoparticles. From the XRD, the unassigned peaks in the vicinity of the characteristic peaks might have resulted from the phytoconstituent capping quercetin on the Au–Ag nanoparticle [32]. Figure 1b depicts a TEM image; the biosynthesized Au–Ag NPs were predominantly partially spherical and triangular in shape. The nanoparticles were covered with organic molecules in red onion extract that stabilized the particles, and Figure 1c shows a Gaussian statistical distribution of the nanoparticles from which the average diameter was estimated to be 30 nm.

### 3.2. UV–Vis Spectroscopy

**Figure 2:**
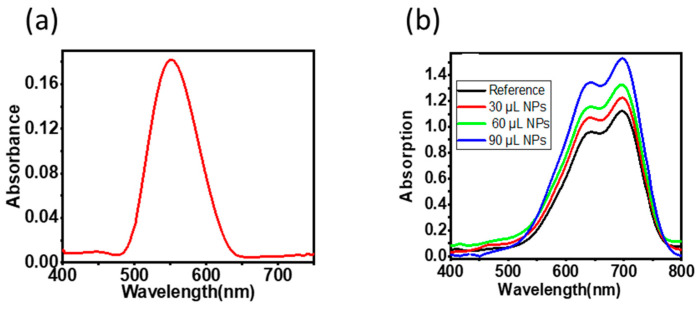
(**a**) The absorption spectrum of Au–Ag NPs solution; (**b**) the absorption spectra of ITO/PEDOT: PSS + Au–Ag/PTB7-Th: ITIC films as a function of the concentration of Au–Ag NP in the PEDOT: PSS.

The Au–Ag NPs optical behavior was investigated by UV–Vis spectroscopy [33,34]. Figure 2a shows the absorption spectra of the Au–Ag NPs. The absorption band at 551 nm is a result of the surface plasmon resonance (SPR) of the nanoparticles. This confirms that the synthesis of the biosynthesized nanoparticles in the aqueous solution and the SPR was at the right frequency. The formation of the band around the region of Au nanoparticles indicates that there was more bio reduction of Au than Ag during the synthesis of Au–Ag NPs [35]. The effect of the synthesized nanoparticles on the active layer was investigated by measuring the absorption spectra of a reference and ITO/PEDOT: PSS/PTB7-Th: ITIC film as a function of the concentration of Au–Ag NPs in the PEDOT: PSS, as shown in Figure 2b. We observed no major changes in the shape of absorption spectra for the different concentrations but a monotonous increase in the overall absorption was observed as the concentration increased. This indicates that the absorption enhancement is probably due to scattering and not due to localized surface plasmon resonance (LSPR) as was previously reported [3,36,37].

### 3.3. Device Performances for Au–Ag NPs Organic Solar Cells

**Figure 3:**
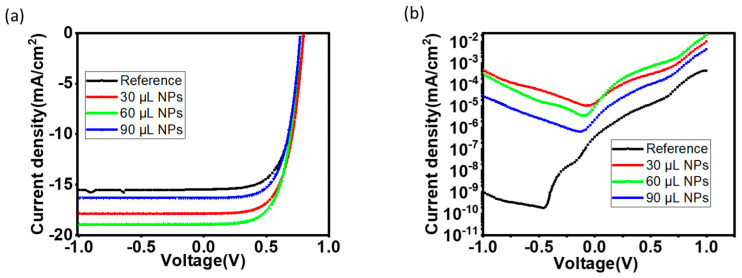
(**a**) J-V characteristics, recorded under illumination at 100 mW cm^−2^ (AM 1.5G) of devices with incorporated Au-Ag NPs in the PEDOT: PSS at different concentrations; (**b**) semi-log of the absolute value of the J-V characteristics in the dark.

Figure 3a,b shows the J-V characteristic curve of the solar device with different concentrations of Au–Ag nanoparticles in PEDOT: PSS and that of reference, i.e., no particles, under illumination and in the dark, respectively.

Figure 3a shows that there is an optimal value for the density of the Au–Ag nanoparticles where the efficiency is maximized. It is clear that the dominant change is in the short circuit current. From this graph, we can also obtain the values of the PCE as reported in the bottom of Table 2. In order to analyze the data further, the measured data, both in the dark and under 1 sun was fitted to a non-ideal single diode model [38] of a solar cell using MATLAB’s fminsearch function. The parameters extracted are series resistance $\left(R_{S}\right)$, photocurrent ($J_{\mathrm{ph}}$), dark saturated current ($J_{S}$), and ideality factor (n). The device parameters obtained from Figure 3a,b are listed in Table 2. The fitting was performed separately for the dark and under illumination measurements and the variations between the two fitted parameters were less than 5%.

From Table 2, it is obvious that power conversion efficiency (PCE) improved with increasing biosynthesized Au–Ag NPs concentration in PEDOT: PSS, reaching a maximum at 60 μL of the nanoparticle’s concentration with a PCE of 9.11%, which corresponds to a 24.7% improvement. This improvement is due to the increase in current density ($J_{\mathrm{sc}}$) and fill factor (FF), from 15.53 to 18.94 mA/cm^2^ and 60 to 62.50%, respectively. From the literature, the addition of nanoparticles to the PEDOT: PSS suppresses the electrostatic interaction between PEDOT and PSS when the nanoparticles form bonds with the sulfur atom of PEDOT, resulting in a substantial PEDOT segregation from the PSS; consequently, the work function of the PEDOT: PSS is changed [8]; the modification of the PEDOT: PSS work function by the addition of Au–Ag nanoparticles could be responsible for the increase in current density [18]. In addition, the behavior of the series resistance ($R_{S}$) that decreases to a minimum value at 60 can also be attributed to the reduced work function of the PEDOT: PSS by Au–Ag NPs which reduces the resistance of the cell [39]. A detrimental effect on the performance of the cells was observed when the concentration of the nanoparticles was further increased to 90 μL with PCE dropping to 7.5%. From the dark I-V in Figure 3b and Table 1, there is a significant difference in the ideality factor (n), at the concentration of 60 μL NPs in PEDOT: PSS; the ideality factor is reduced to 2.91, which suggests less recombination. This is further supported by a reduced value of dark saturation current density ($J_{S}$) to $7.32667\times \;10^{-7}$ mA/cm^2^. However, further increment of the nanoparticles’ concentration (90 μL NPs) leads to an increase in the ideality factor and dark saturation current to 3.1141 and $1.1\times \;10^{-6}$ A/cm^2^, respectively. The increase in recombination could be a consequence of the aggregation of nanoparticles at 90 μL, leading to trapping and quenching of excitons and resulting in low PCE [40].

#### Au–Ag NPs Effect on Exciton Quenching

From Table 2, the reference sample has a photocurrent of 0.0156; incorporating Au–Ag NPs in the PEDOT: PSS increases the photocurrent to 0.0189 at 60 μL NPs. Further increase in the nanoparticle concentration to 90 μL leads to a decrease in photocurrent and a subsequently degraded PCE. Initially, this seems to contradict the UV–Vis spectra in Figure 2b, where the absorption intensity increases continuously with an increase in nanoparticle concentration. According to the literature’s report on photoluminescent analysis on ITO/PEDOT: PSS: NPS/active layer film [38], two reasons may be responsible this phenomenon: changes in optical absorption due to LSPR, and metal–organic interface exciton quenching [41,42]. The effect due to LSPR is neglected, as we have experimentally demonstrated in Figure 2b. We therefore attribute the reduction in the photocurrent to exciton quenching as predicted by the CPS model [43,44]. To study the effect of the synthesis procedure on this behavior is a topic for future research as it has been reported that an organic capping agent on the Au–Ag NPs’ surface can act as an insulating layer, thus preventing direct contact between the metal and the active layer and reducing exciton quenching on the surface of biosynthesized Au–Ag nanoparticles [45].

**Figure 4:**
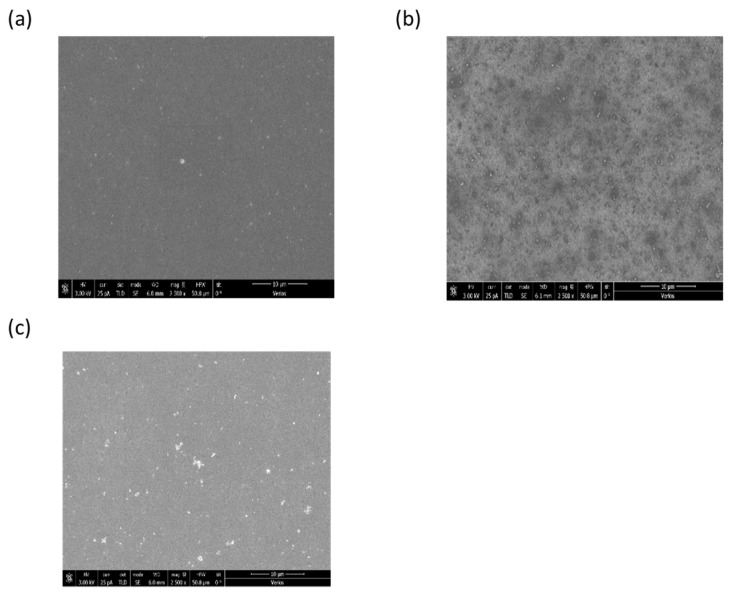
SEM images of the surface of PEDOT: PSS: Au–Ag NPs films with (**a**) PEDOT: PSS with 30 μL NPs; (**b**) PEDOT: PSS with 60 μL NPs; and (**c**) PEDOT: PSS with 90 μL NPs.

To further understand the influence of the concentration on the photocurrent, we studied, using SEM, the morphology of the PEDOT: PSS + Au–Ag NP as a function of the NP concentration. The results are shown in Figure 4 below.

Figure 4 shows the morphology of PEDOT: PSS: Au–Ag NPs film as a function of different nanoparticle concentrations using SEM; with 30 μL NPs in PEDOT: PSS, very few particles were seen. When the concentration was increased to 60 μL NPs, we can clearly observe more particles with good dispersion. From the literature, the morphological change could increase the anode surface roughness, resulting in an increase the interface area between the active layer and the anode, creating a shorter way for the hole to move to the anode and enhancing anode hole collection [46]. This is an additional possible explanation to the observed increase in $J_{\mathrm{sc}}$ as seen in Table 2. However, at a higher concentration of Au–Ag NPs (90 μL), a deteriorating effect was observed as discussed above. This can be attributed to the aggregation of nanoparticles, as shown in Figure 4c, resulting in a poor intermixing of the blend component, causing an increase in the exciton quenching and the recombination of electron-hole pairs due to reduced charge separation or transfer.

## 4. Conclusions

In conclusion, by incorporating biosynthesized Au–Ag nanoparticles in PEDOT: PSS, a 24.7% improvement in power conversion efficiency (PCE) from 7.3% to 9.11% for non-fullerene acceptor-based organic solar cells was observed. This enhancement is clearly related to the increase in the short circuit current density (Jsc) and fill factor (FF). The highest PCE performance is obtained at a 60 μL concentration of biosynthesized Au–Ag nanoparticles. The increase in the short circuit current is attributed to an increase in the photocurrent due to scattering by the Au–Ag NPs. We have excluded the possible contribution of the LSPR effect because there is no absorption shift between the reference and the sample with Au–Ag NPs. Moreover, SEM images of the morphologyy of the surface of the PEDOT: PSS for different NP concentrations show an increase in the roughness of the anode surface, resulting in an increase in the interface area between the active layer and the anode, thus creating a shorter path for the hole to move to the anode and enhancing the anode hole collection. The enhanced hold transport is responsible for the reduction of the series resistance and improved filling factor. The reduction in the cell performance when the NP concentration is above 60 μL is attributed to exciton quenching at the metal–organic interfaces as the NP coalesce with the increased concentration. This effect might not be seen at lower concentrations as it has been reported that an organic capping agent on the Au–Ag NPs surface acts as an insulating layer which prevents direct contact between the metal and active layer, leading to a reduction in the exciton quenching on the surface of the biosynthesized Au–Ag nanoparticles which possibly manifests itself in a reduced ideality factor. Future study will focus on trying to create the NPs in situ within the PEDOT: PSS, thus minimizing the number of required steps.

## Figures and Tables

**Table 1:** Comparisons of power conversion efficiency of organic solar cells with and without nanoparticles incorporated in the different parts of the device.

Device Configuration	NPs	Position	PCE %		Ref.
			W/O NPs	W/NPs	
ITO/PEDOT: PSS/P3HT: PCBM/LiF/Al	Ag: Mg	P3HT: PCBM	2.29	4.11	[5]
ITO/PEDOT: PSS/P3HT PCBM/Al	Ag/Ag_2_O	PEDOT: PSS	3.06	5.20	[6]
ITO/PEDOT: PSS/PTB7: PC_71_BM/Al	Au–Ag	PEDOT: PSS/PTB_7_: PC_71_BM	2.50	4.24	[7]
ITO/PEDOT: PSS/PBDB-T: ITIC/PFN/FM	Ag	PEDOT: PSS	5.9	6.4	[8]
ITO/ZnO/PTB7-Th: PC71BM/MoO_3_/Al	Au	ZnO	9.31	11.8	[9]
ITO/PEDOT: PSS/P3HT: PCBM/LiF/Al	Au	P3HT: PCBM	2.11	2.55	[10]
ITO/PEDOT: PSS/P3HT: PCBM/LiF/Al	Ag	P3HT: PCBM	2.11	2.23	[10]
ITO/PEDOT: PSS/PCPDTBT: PC_60_BM/Al	Ag	PEDOT: PSS	0.0057	0.0396	[11]
ITO/PEDOT: PSS/P_3_HT: PCBM/Al	Au	PEDOT: PSS	3.45	3.65	[12]
ITO/PEDOT: PSS/P3HT: PCBM/Al	Au	PEDOT: PSS	2.963	3.510	[13]
ITO/ZnO/PTB7: PCBM/MoO_3_/Ag	Ag	ZnO	6.53	7.25	[14]
ITO/PEDOT: PSS/CuPc/C60/Al	Au	PEDOT: PSS	0.78	1.02	[15]
ITO/PEDOT: PSS PBDB-T-2Cl: IT-4F/PDINO/Al	Ag	PEDOT: PSS	12.85	13.42	[16]
(ITO)/PEI/P3HT: ICBA)/WO_3_/WO_3_/Ag	Ag–Au	WO_3_	4.57	6.55	[17]
ITO/PEDOT: PSS/P3HT: PCBM/Al	Au	PEDOT: PSS	2.44	2.88	[18]
ITO/PEDOT: PSS/PTB7-Th: ITIC/PFN/Al *	Au-Ag	PEDOT: PSS	7.3	9.11	

* This work.

**Table 2:** Summary of the photovoltaic performance of the organic solar cells with different concentrations of the Au–Ag nanoparticles in the PEDOT: PSS: experimental and MATLAB simulation.

Sample	J_SC_ (mA/cm^2^)	V_OC_ (V)	FF (%)	PCE (%)	n	J_S_ (mA/cm^2^)	R_S_ (Ωcm^2^)	J_ph_ (mA/cm^2^)
Reference	15.53	0.79	60.0	7.30	3.33	1.04 × 10^−6^	2.99	0.0156
30 μL NPs	17.87	0.79	61.0	8.63	3.4	9.87 × 10^−7^	2.15	0.0180
60 μL NPs	18.94	0.77	62.5	9.11	2.91	7.33 × 10^−7^	1.74	0.0189
90 μL NPs	16.29	0.77	60.2	7.55	3.21	1.08 × 10^−6^	2.37	0.0165

## Data Availability

Not applicable.
